# The Role of Socio-Affective and Socio-Cognitive Mechanisms in the Processing of Witnessed Traumatic Events

**DOI:** 10.3389/fpsyt.2022.830218

**Published:** 2022-03-10

**Authors:** Sebastian Trautmann, Charlotte Wittgens, Markus Muehlhan, Philipp Kanske

**Affiliations:** ^1^Department of Psychology, Faculty of Human Sciences, Medical School Hamburg, Hamburg, Germany; ^2^ICPP Institute of Clinical Psychology and Psychotherapy, Medical School Hamburg, Hamburg, Germany; ^3^ICAN Institute for Cognitive and Affective Neuroscience, Medical School Hamburg, Hamburg, Germany; ^4^Institute of Clinical Psychology and Psychotherapy, Faculty of Psychology, Technische Universität Dresden, Dresden, Germany

**Keywords:** empathy, theory of mind, hyperarousal, negative thinking, traumatic event

## Introduction

Experiencing traumatic events has a high lifetime prevalence ranging between 60.7 and 76.2% across different countries (1). Exposure to traumatic events is associated with a higher risk for various mental disorders such as posttraumatic stress disorder (2, 3), which are related to high individual and societal costs (4). The development of interventions to prevent adverse mental health consequences following traumatic event exposure is therefore of vital importance. This, however, requires detailed knowledge about the underlying biological and psychological mechanisms involved in the association between traumatic events and psychopathology. Various risk factors at different levels have already been described in the last decades (5). Biological risk factors include genetic and epigenetic variations (6), alterations in the function of the hypothalamic pituitary adrenal (HPA) axis (7, 8) and the autonomic nervous system (9) as well as changes in brain structure and functioning (10). Psychological risk factors include impairments in cognitive abilities (11) and specific personality traits such as high trait anxiety (12) and maladaptive emotion regulation (13). Social risk factors include impaired interpersonal relations and stigmatization (14, 15). Further, clinical risk factors such as mental health history as well as previous traumatic experiences may also increase the risk for psychopathology after trauma exposure (16). Most of these factors are supposed to be associated with risk of psychopathology independent of the type of traumatic event. However, it is likely that specific traumatic events are associated with different constellations of risk factors, which has so far received little attention in the existing literature. Importantly, traumatic events explicitly include not only events that are personally experienced but also events that are witnessed by an observer (17). This includes witnessing someone being seriously hurt, seeing atrocities or witnessing dead bodies. Witnessed traumatic events are among the most frequent traumatic experiences (1). They are also of high current relevance in the contexts of natural disasters, terrorist attacks and military crises (16, 18, 19). The fact that individuals can develop psychopathological reactions to events that are actually experienced by others raises the question how the suffering of others is being processed. Based on theoretical models and findings from social cognition and neuroscience research, we propose that socio-affective and socio-cognitive mechanisms are involved in the processing and pathological consequences of witnessing traumatic events and could contribute to a better understanding of adverse reactions to this type of traumatic events.

## The Role of Socio-Affective and Socio-Cognitive Mechanisms in the Processing of Witnessed Traumatic Events

### The Role of Empathy and Theory of Mind in the Processing of Witnessed Traumatic Events

**Figure 1:**
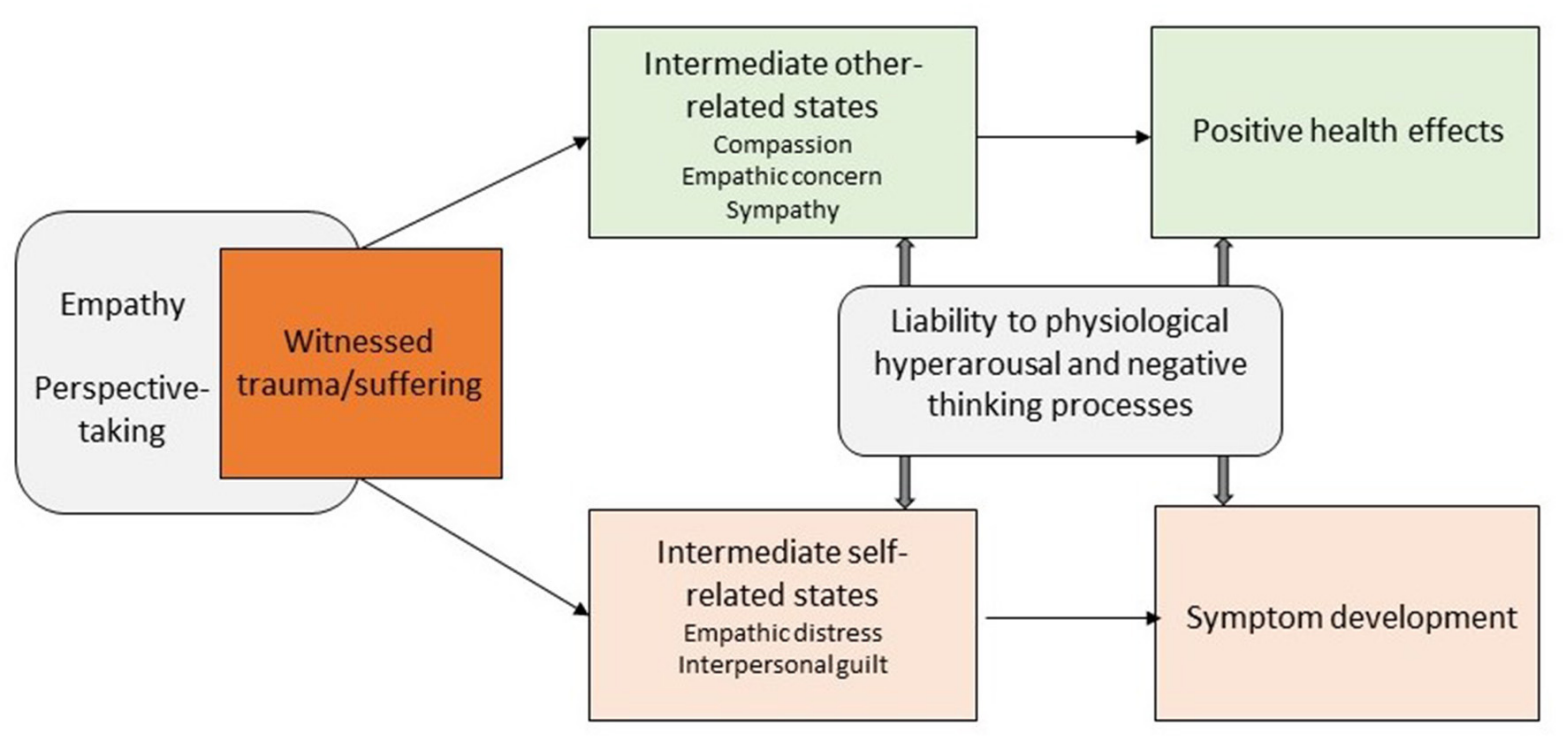
Proposed socio-affective and socio-cognitive mechanism and pathways in the processing of witnessed traumatic events [based on (37, 41)].

There is solid evidence for the tendency to psycho-physiologically resonate with others' stress responses (20). This linkage with an observed individual that is experiencing adverse events can be associated with stress reactions in the observer (21, 22). Importantly, this linkage between target and observer has been closely related to the constructs of empathy and perspective-taking (also referred to as Theory of Mind or mentalizing). Empathy denotes the sharing of another person's emotions and can thus be defined as an affective state in an observer that is isomorphic to an observed person's affective state (23). Perspective-taking enables the reasoning about and understanding of others' mental states, including their emotions (24) and can modulate empathic responding (25). Adaptive social interaction critically depends on these capacities to understand and feel with others and most empirical evidence associates perspective-taking and empathy with positive health outcomes. Empathy is associated with better relationship quality (26), greater professional satisfaction (27), and emotional self-efficacy (28), with all of these factors being associated with positive mental health (29). Empathy is also proposed to be a healthy and efficient method of interpersonal emotion regulation (30). More specifically, there is first evidence that empathy is related to resilience after secondary exposure to traumatic events (31). On the other hand, there is also evidence for a relationship between empathy and adverse mental health symptoms such as depression (32) and anxiety (33). Moreover, empathy was found to be related to secondary trauma in caregivers (34, 35) and physicians (36, 37) and also with higher levels of traumatic stress symptoms (38, 39). Thus, it can be assumed that empathic responding is only an initial processing step that can be followed by diverging socio-affective and socio-cognitive functions, representing two distinct pathways resulting in either negative or positive mental health outcomes (Figure 1).

### The Mediating Role of Self- and Other-Related Intermediate States

As described above, empathy and perspective-taking may lead the observer to feel positive, caring emotions for the suffering other (upper path in Figure 1). These emotions, which form intrinsically other-related states (40), have been studied as compassion, empathic concern or sympathy. Training studies have shown that compassion can be cultivated, increasing not only subjective reports of positive affect toward others, but also prosocial helping behavior (41, 42). As for compassion, it is conceptualized as a qualitatively different state than empathic distress, one that can be actively generated (43). It may be explicitly cultivated as is done, for instance, in compassion focused therapy (44). It may also arise spontaneously, but not automatically when there is no empathic distress (45, 46). Compassion also varies greatly in untrained individuals, with stronger compassionate responding being again associated with more prosocial behavior (47). Furthermore, trait levels of (self-) compassion have been related to stress-buffering and anti-depressant effects (48) and were associated with mental health and recovery from adverse events (49, 50) and with lower PTSD symptoms after witnessed trauma (51). For the context of traumatic stress research, these results suggest that these other-related states may be promising candidates that could partially explain resilience after witnessing of traumatic events.

A second possible outcome of initial perspective-taking and empathic sharing of another's suffering is, however, an elevated risk of adverse reactions including symptom development (lower path in Figure 1). Recent theoretical models (37) suggest that developmental trajectories from empathic tendencies to symptom development encompass two intermediate conditions: empathic distress and interpersonal guilt. In contrast to the other-related states described above, empathic distress and interpersonal guilt are negative states that are intrinsically self-related. Because it shares the negative valence with the initial empathic response to others' suffering, empathic distress may be viewed as an excessive form of empathy and is characterized by increased arousal, stress responses and fear (52). Interpersonal guilt could be viewed as a maladaptive form of cognitive empathy that is driven by excessive concerns, such as unreasonable beliefs that one is responsible for alleviating the suffering of others (53). Both empathic distress and interpersonal guilt may contribute to a higher risk of later psychopathology (41, 54, 55). Thus, in the context of witnessing trauma, empathic distress and interpersonal guilt may be important mediators of adverse reactions and psychopathological symptoms.

### The Moderating Role Empathic Sensitivity, Physiological Hyperarousal, and Negative Thinking Processes

The association between empathy and mental health is likely to be non-linear with moderate levels being related to beneficial and high levels to adverse outcomes (37). In addition, previous research suggests that the outcome of empathic responding is further moderated by (1) liability to physiological hyperarousal and (2) liability to negative thinking processes (see Figure 1). Liability to high physiological arousal characterized by alterations in basal endocrine (e.g., basal cortisol secretion) and autonomic changes (e.g., heart rate variability) has been associated with symptom development after exposure to direct and observed stressful experiences (7, 56, 57). Liability to negative thinking processes includes the predisposition to self-focused rumination and poor regulation of cognitive processes (e.g., cognitive inflexibility, impaired ability to suppress negative thoughts) conceptualized as stable traits. They have been associated with poor coping and symptom development in the context of stress exposure and witnessed trauma (58–60). Taken together, these findings suggest that empathic distress and its adverse consequences might be the result of a liability to empathic sensitivity which interacts with a liability to physiological hyperarousal and negative thinking processes.

## Investigating Socio-Affective and Socio-Cognitive Mechanisms in the Processing of Witnessed Traumatic Events

A sound investigation of potential socio-affective mechanisms of witnessed trauma including its moderators requires experimental designs to be able to manipulate the independent variable (witnessed trauma) and to take into account the existence of potential confounding factors. In recent years, laboratory models of witnessed trauma such as the trauma film paradigm have been developed and successfully implemented in various studies (61). However, it must be considered that the external validity of such trauma analog studies is limited. Therefore, there is a need to test hypotheses also with other study designs such as prospective cohort studies or cohort studies in recently trauma exposed individuals. To elucidate which factors contribute to either beneficial or adverse pathways of empathic processes after witnessed trauma, a social cognitive and affective neuroscience approach could also be particularly valuable (62). Perspective-taking, empathy, compassion and empathic distress are dissociable on interindividual, intraindividual developmental and neural levels (63, 64). Perspective-taking activates regions in the temporoparietal junction and anterior and posterior midline structures (65), while different networks are involved in sharing different emotions. For sharing others pain and negative emotions in general, it is especially the anterior insula, anterior cingulate cortex, and amygdala that are involved (46). Empathic distress may include activation change in the anterior insula and cingulate cortex, but also in the amygdala and hippocampus as has been shown for first-hand stress experience (66, 67). Compassion, in contrast, activates a network typically involved in positive affect and reward processing including the ventral striatum and medial orbitofrontal cortex (47). Probing the neural responding to witnessing traumatic events would enable the objective assessment of perspective-taking, empathic affect sharing, compassion and empathic distress as potential predictors of later symptom development. In addition to neuroimaging methods, there are well-validated and reliable paradigms for behavioral assessments of socio-cognitive and socio-affective processes such as compassion, empathic concern and perspective-taking (theory of mind) (68). For instance, the EmpaToM task presents videos of short autobiographic narrations that vary in emotion and perspective-taking demands (68, 69). These enable the assessment of socio-affective and -cognitive functioning with meaningful variability in health and psychopathology that also relates to everyday functioning (70, 71). Lastly, genetic contributions might be a valuable target to explain differences in empathic sensitivity (37) and could serve as potential biological risk markers.

## Summary and Conclusions

Witnessed traumatic events are highly prevalent and can cause high individual and societal burden. In addition to known risk factors for symptom development, socio-affective, and socio-cognitive mechanisms could play a crucial role for the processing of such events. Perspective-taking and empathic responding are initial processing steps, followed by diverging socio-affective functions, which are associated with either negative or positive affective and health outcomes. Although these proposed trajectories are still merely theoretical and evidence supporting the specificity of the suggested mechanisms beyond known concepts of risk and resilience is scarce, they present highly valuable targets for future research. Confirming different socio-affective pathways, their dissection on the neural level and the identification of biological and psychological factors that contribute to these different pathways could improve the prediction of adverse reactions to witnessed trauma.

## Author Contributions

ST developed the concept and wrote the paper. CW contributed to the writing of the paper and revised the paper for important intellectual content. MM revised the paper for important intellectual content. PK contributed to the concept and revised the paper for important intellectual content. All authors approved the final manuscript for publication.

## Funding

ST and PK are supported by the German Research Foundation (TR1489/1-1, KA 4412/2-1, KA 4412/4-1, KA 4412/5-1, and CRC940/C07).

## Conflict of Interest

The authors declare that the research was conducted in the absence of any commercial or financial relationships that could be construed as a potential conflict of interest.

## Publisher's Note

All claims expressed in this article are solely those of the authors and do not necessarily represent those of their affiliated organizations, or those of the publisher, the editors and the reviewers. Any product that may be evaluated in this article, or claim that may be made by its manufacturer, is not guaranteed or endorsed by the publisher.
